# Author self-citations in the urology literature

**DOI:** 10.1080/2090598X.2022.2056976

**Published:** 2022-03-30

**Authors:** Vaibhav Aggarwal

**Affiliations:** Department of Surgical Disciplines, All India Institute of Medical Sciences (AIIMS), New Delhi, India

**Keywords:** Author self-citations, bibliometrics, diachronous self-citations, negative binomial regression

## Abstract

**Objective:**

We aimed to determine the diachronous self-citation rate and the various article characteristics which can influence the rate and percentage of diachronous author self-citations using papers published in high-rank urology journals.

**Methods:**

We included all papers (N = 327 articles) published between January 2015 to April 2015 in the European Urology, The Journal of Urology and the BJU International. We determined author self-citations using the Scopus database and used negative binomial regression to determine which article characteristics affect self-citations.

**Results:**

262 articles (80.2%) contained at least one self-citation.The mean number and percentage of author self-citations were 6.5 and 14.2 respectively. Adjusted analysis showed that the experimental/animal study design and the number of authors were significantly associated with both the number (IRR = 2.12, P = 0.011; IRR = 1.03, P = 0.002) and percentage of author self-citations (IRR = 2.95, P = 0.003; IRR = 1.03, P = 0.012). The number of citations in the Scopus and publication in European Urology were significantly associated with only the number of author self-citations.

**Conclusion:**

Diachronous author self-citation rate in urology is higher compared to general medical literature but similar to other surgical subspecialties. It may depend on the study design and the number of authors in the paper. For a more comprehensive evaluation, future studies should look at the context in which self-citations were made.

## Introduction

The quest to publish in academia has grown significantly over the years and academic surgery is no exception. Nonetheless, This is much needed to fill in the evidence gaps to guide decision making, deliver the best possible quality of care and reduce variations in surgical practice [1]. Academically productive surgeon scientists are often preferred for hiring and promotions to major academic positions. Scholarly productivity is usually assessed by the number of publications, grant funding or citation based metrics such as the number of citations and h-index [2]. However, citation-based metrics are prone to manipulation through self-citations [3]. This practice could be misleading and can potentially distort the scientific literature [4].

Lawani has defined 2 genera of self-citations: synchronous (the ones which are contained in the reference list of the indexed article) and diachronous (the ones which are contained in the citations the indexed article receives) [5]. Previous studies have tried to quantitatively measure the impact of author self-citations on citation metrics in general medicine [6], plastic surgery [7], otolaryngology [8], orthodontics [9] and radiology [10]. However, Little is known about diachronous author self-citations rates in surgical subspecialties such as urology. In addition, it was found that among surgical subspecialties, urologists and neurosurgeons had a considerably higher number of h-index [11]. The reasons for such differences are not clear. Moreover, article characteristics on which self-citations depend have not been adequately evaluated in the surgical literature.

This study aimed to determine the diachronous self-citation rate and the various article characteristics which can influence the rate and percentage of diachronous self-citations using papers published in a 4 month period in the 3 high ranking urology subspecialty journals.

## Methodology

### Study design and data collection

As the study did not involve human participants, research ethics committee approval was not applicable. This is a retrospective study that included articles published between January 2015 to April 2015 in the European Urology (EU), The Journal of Urology (JU) and BJU International (BJUI) through hand searching. These were chosen based on their importance in urology according to the SCImago Journal Rank indicator. These journals were accessed through institutional access.

The articles were subdivided by study design such as systematic reviews/ meta-analysis/ reviews, interventional studies, prospective observational studies, retrospective studies/ case series/ case reports and experimental/ animal studies. Articles types such as abstracts, editorials, and meeting reviews were excluded.

In April 2021, after 6 hours of training in the use of Scopus at the institute’s library, the author extracted certain article characteristics from each of the above-selected articles as described below. The author randomly selected 40 articles to confirm the accuracy of data collection.

For each article we extracted the following characteristics:

1. The journal in which the article appeared (EU, JU, BJUI)
2. Study sub-topic
3. The study design (experimental/ animal studies; guidelines; case reports/ case series/ retrospective studies/ cross sectional studies; prospective observational studies; interventional studies and reviews/ systematic review/ meta-analysis)
4. The month of publication (January 2015 to April 2015)
5. Country of the corresponding author
6. Total number of authors
7. Number of characters in the title (excluding trailing or double spaces)
8. The total number of citations in the Scopus database (this includes the absolute number of citations received by publications until April 2021).
9. Whether the research was funded.
10. Whether the article is an open access

## Dependent variables

1. Number of author self-citations in Scopus database
2. Percentage of author self-citations in Scopus database (determined by the proportion of author self-citations to the total number of citations received by the article)

### Statistical analysis

**Figure 1.**
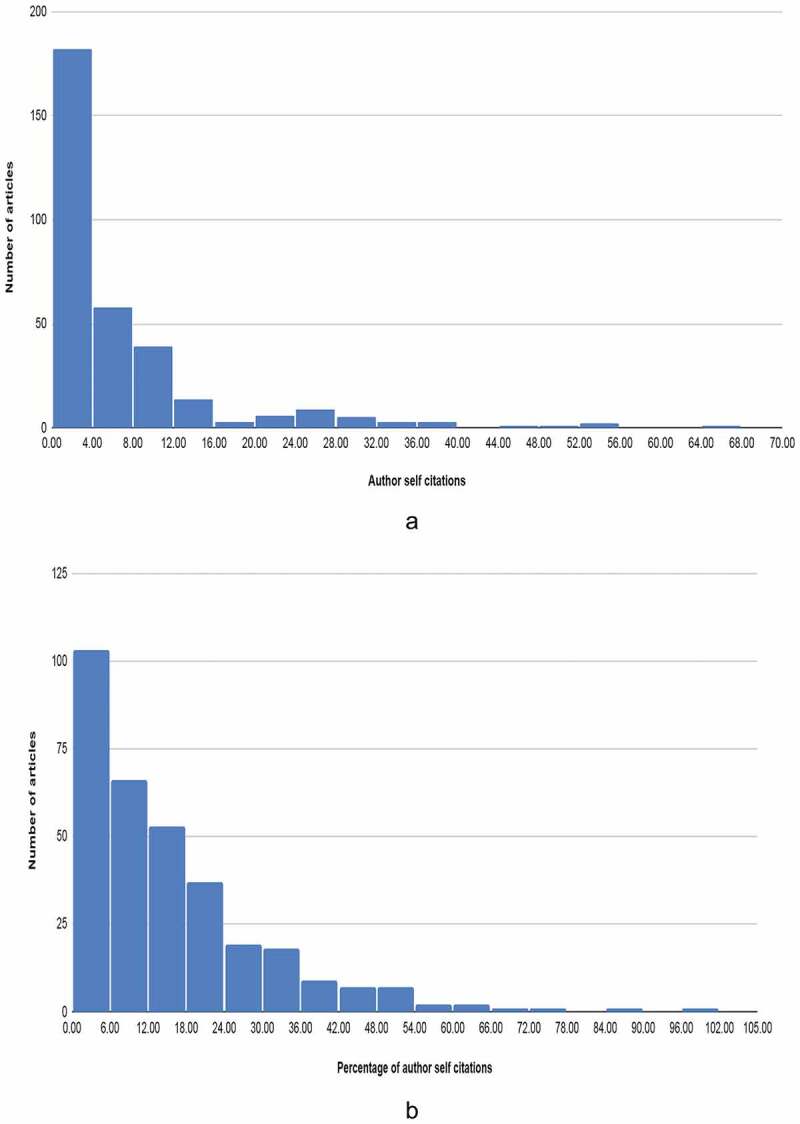
A. Frequency distribution of number of author self citations. B. Frequency distribution of percentage of author self citations. The distributions are right skewed and follow a negative binomial pattern.

We inserted all the data for each included article into spreadsheet software. This database was then analysed using IBM SPSS Statistics v26. Descriptive statistics were used to quantitatively describe the features of the sample. The variance in the number of self-citations, and the percentage of author self-citations in this study were much greater than their mean signifying overdispersion. In addition, these variables did not have an excessive number of zero values. Therefore, we rejected the Poisson regression and the zero-inflated models. We selected the negative binomial regression model to determine the effect of the article characteristics on self-citations (Figure 1). To transform the percentage of author self-citations into count data, we rounded off the percentage to the nearest whole number. We included the following independent variables in the model: 1) The journal in which the article appeared; 2) The study design; 3) Study sub-topic; 4) Country of the corresponding author; 5) Whether the research was funded; 6) Whether the article is an open-access; 7) Number of characters in the title; 8) The total number of authors; 9) Number of references; 10) The total number of citations in the Scopus database.

The analysis was performed on the entire study cohort. There were no missing values in the data set. All statistical tests were two-sided, and P values of less than 0.05 were considered to indicate statistical significance.

## Results

Overall, 327 articles were included in the analysis. Characteristics of the included articles are shown in Table 1. Of these, EU, JU and BJUI constituted 21.7%, 53.5% and 24.8% of total articles respectively. 37.3% of the articles received funding and 30% of the articles were open access.‘Prostate’ was the most studied topic (35.8%). Retrospective studies and case series were the most commonly used study designs. The corresponding author was affiliated with an institution in the United States in 45.9% of the articles. The mean number of title character count, reference count and authors were 121.6 (range 36–275), 28.1 (range 5–80) and 9.2 (range 1–42) respectively.

**Table 1.** Baseline characteristics*.

**Variables**	**Articles (study group)** **(N = 327)**
Journal European Urology Journal of Urology BJU International	71 (21.7) 175 (53.5) 81 (24.8)
Month of publication January 2015 February 2015 March 2015 April 2015	83 (25.4) 79 (24.2) 77 (23.5) 88 (26.9)
Study design Experimental/ Animal studies Guidelines Case reports/ case series/ retrospective studies/ cross sectional studies Prospective observational studies Interventional studies Reviews/ Systematic review/ meta-analysis	31 (9.5) 6 (1.8) 172 (52.6) 41 (12.5) 40 (12.2) 37 (11.3)
Country of the corresponding author Australia Canada United Kingdom United States France/Germany/Netherlands/Italy others^#^	7 (2.1) 22 (6.7) 30 (9.2) 150 (45.9) 52 (15.9) 66 (20.2)
Topic Renal Ureter Bladder Prostate Urethra/penis Sexual/reproductive Miscellaneous	46 (14.1) 7 (2.1) 64 (19.6) 117 (35.8) 17 (5.2) 20 (6.1) 56 (17.1)
Funding Yes No	122 (37.3) 205 (62.7)
Open Access Yes No	98 (30) 229 (70)
Character count of the title	121.6 ± 41.4
Number of authors	9.17 ± 5.8
Reference count	28.12 ± 12.8
Citation count in Scopus database	45.94 ± 54.2
Citations per year in Scopus database	7.7 ± 9
Self citation count in Scopus database	6.54 ± 9.6
Percentage of author self citations	14.2%

*Plus-minus values are means ±SD. Number in parenthesis denotes percentages.

# Denote these countries: Brazil/ Belgium/ Sweden/ Spain/ South Korea/ Denmark/ China/ Japan/ Switzerland/ India/ Egypt/ Greece/ Ireland/ Turkey/ New Zealand/ Norway/ Portugal/ Singapore/ Taiwan

The mean number of citations and citations per year received by an article in the Scopus database was 45.9 (range 1–411) and 7.7 (range 0.16–68.5). 262 articles (80.2%) contained at least one self-citation. The mean number of author self-citations was 6.5 (range 0–65). The percentage of author self-citations (calculated from the proportion of the sum of author self-citations of all the included articles with the sum of all citations of all the included articles) was 14.2%.

### Negative binomial regression analysis

The variance inflation factor (VIF) was calculated for each variable included in the model. All VIF values were <3 showing that there was no multicollinearity. Therefore, all variables were considered in the regression analysis. Adjusted analysis showed that experimental/animal study design was statistically significantly associated with both the number of author self-citations (incidence rate ratio (IRR) = 2.12; 95% confidence interval (CI) = 1.2 to 3.77; P = 0.011) and with percentage of author self citations (IRR = 2.95; 95% CI = 1.46 to 5.96; P = 0.003). Similarly, the number of authors in an article was strongly correlated with both the number of author self-citations (IRR = 1.03; 95% CI = 1.01 to 1.05; P = 0.002) and with percentage of author self citations (IRR = 1.03; 95% CI = 1.007 to 1.06; P = 0.012).

Publication of an article in European Urology and the total number of citations in the Scopus database was also statistically significantly associated with the number of author self-citations (IRR = 1.67; 95% CI = 1.14 to 2.45; P = 0.009 and IRR = 1.01; 95% CI = 1.01 to 1.02; P = 0.000) but was not associated with the percentage of author self-citations. It is important to note that these isolated significant results on the number of self citations from regression analysis may just be due to higher citation counts. No statistically significant association was found with other variables included in the regression (Table 2).

**Table 2.** Negative binomial regression IRR for the outcomes.

**Variable**	**Number of author self citations**	**Percentage of author self citations**
Journal European Urology Journal of Urology BJU International	1.17 (1.14–2.45) 1.27 (0.93–1.7) 1	1.48 (0.9–2.41) 1.25 (0.87–1.8) 1
Study design Experimental/ Animal studies Guidelines Case reports/ case series/ retrospective studies/ cross sectional studies Prospective observational studies Interventional studies Reviews/ Systematic review/ meta-analysis	2.12 (1.19–3.77) 1.27 (0.55–2.94) 1.49 (0.93–2.37) 1.56 (0.92–2.65) 1.23 (0.7–2.16) 1	2.95 (1.46–5.96) 1.49 (0.51–4.34) 1.67 (0.94–2.94) 1.68 (0.88–3.21) 1.38 (0.71–2.69) 1
Country of the corresponding author Australia Canada United Kingdom US France/Germany/Netherlands/Italy others^#^	1.4 (0.64–3.05) 0.91 (0.56–1.5) 0.95 (0.6–1.5) 1.04 (0.76–1.42) 1.22 (0.83–1.8) 1	1 (0.37–2.68) 0.74 (0.4–1.36) 0.87 (0.49–1.54) 0.89 (0.6–1.31) 0.99 (0.6–1.62) 1
Topic Renal Ureter Bladder Prostate Urethra/penis Sexual/reproductive Miscellaneous	0.82 (0.54–1.24) 0.8 (0.36–1.84) 1.07 (0.73–1.56) 1.06 (0.75–1.49) 1.03 (0.59–1.79) 0.86 (0.49–1.49) 1	0.74 (0.45–1.21) 0.7 (0.26–1.9) 1.1 (0.7–1.73) 1.06 (0.7–1.6) 1.06 (0.54–2.06) 0.91 (0.48–1.76) 1
Funding Yes No	0.98 (0.76–1.27) 1	0.93 (0.68–1.28) 1
Open Access Yes No	1.16 (0.88–1.53) 1	1.08 (0.78–1.5) 1
Character count of the title	1 (0.99–1.0)	0.999 (0.996–1.003)
Number of authors	1.03 (1.01–1.05)	1.03 (1.007–1.06)
Reference count	1 (0.99–1.01)	1.0 (0.99–1.02)
Citation count in Scopus database	1.014 (1.011–1.017)	0.998 (0.995–1.001)

*IRR stands for incidence rate ratios. Values in the table are expressed as IRR (95% confidence interval)

# Denote these countries: Brazil/ Belgium/ Sweden/ Spain/ South Korea/ Denmark/ China/ Japan/ Switzerland/ India/ Egypt/ Greece/ Ireland/ Turkey/ New Zealand/ Norway/ Portugal/ Singapore/ Taiwan.

## Discussion

In this retrospective study of 327 articles, evaluating the impact of various articles characteristics on author self-citation counts, we found that the overall percentage of self-citation in urology literature was 14.2% with a median of 3. Following article characteristics were associated with a higher number of self-citations: experimental/ animal studies, higher number of authors, publication of an article in EU and the total number of citations in the Scopus database. Similar results were obtained for experimental/animal studies and higher number of authors when considering the percentage of author self-citations as the outcome. However, publication in the EU and the total number of citations were not found to be significantly associated.

The rate of self-citations across various disciplines of medicine has been variable. Kulkarni et al. report that it amounts to 7% in the general medical literature [6]. However, the self-citation rate is higher in subspeciality literature including diabetes [12], otolaryngology [8], cardiovascular medicine, infectious diseases [6] and musculoskeletal radiology [10] similar to the one in our study. This is true for journal self-citations as well such as dermatology [13] and orthopaedics [14]. Moreover, we found that experimental/ animal studies are associated with higher self-citations similar to observations made by authors of otolaryngology literature [8]. This is expected since expertise in subspecialized fields may be limited. In addition, science built on basic hypotheses is more likely to inspire further research by the same group. When such expertise is coupled with novel research questions and a limited number of journals to publish in, self-citations become inevitable.

The number of authors was independently significantly associated with higher self-citations. Previous studies on self-citations have yielded similar observations [6] [8]. We can anticipate this however since more authors are available to self cite. This might also help increase the interdisciplinary visibility and influence of the paper, which may attract more total citations and self-citations. Studies conducted on ‘citation classics’ in surgery and its subspecialties consistently show that western and European countries produce the most number of top-cited papers [15] [16]. But this did not translate to a high self-citation rate in our study and other similar studies. This may be because literature considered ‘important’ in the field does not require self promotion to be well recognized by peers irrespective of the topic of study. This argument is further strengthened by the finding that the percentage of author self-citations was slightly negatively correlated with the number of non-self citation counts in Scopus that reached statistical significance (r = −0.13, P = 0.018). In addition, some data indicate that the self-citation rate may be higher in papers that are poorly cited [17]. Thus the rate and percentage of self-citations remained similar and statistically non-significant across nations and the topic of the study.

Self-citations could be viewed as a double edged sword. On one hand, they are healthy for scientific research and are needed (and sometimes even unavoidable) to network with previously established concepts or observations and aids in understanding and expanding on research hypotheses. This is how Mishra et al [18]. justifies the existence of self-citations, ‘lack of (self-)citations likely reflects dead-ends and orphans not even nurtured by the scholars with a vested interest.’ However, when manipulative, self-citations may propagate a specific thought process or methodology which may be biased and unscientific. It may also be able to create a false perception of the importance or authority of an individual in the field particularly in a subspecialty where there are a limited number of well-cited authors.

We used robust statistical methods in this paper. We determined which article characteristics influence self-citation in subspecialty urosurgery literature using a sufficiently long publication window and by adjusting for potential confounders. There are limitations to this study, however. We used a retrospective study design which has an inherent potential for unknown confounding. Furthermore, this study included articles from the EU, JU and BJUI which are the high ranking urology subspecialty journals. Subspeciality journals often publish papers having a narrow clinical readership. Thus, these findings may not be generalizable to general medical and surgical journals which have a broad readership and a broad citing community. Similarly, these results may not be generalizable to small journals as they often have a small readership and less often publish highly cited papers. These ‘less prestigious’ journals may have higher self-citation rates [19]. Lastly, though Scopus has a fair accuracy in determining author self-citations, results might vary if other databases are used.

## Conclusion

Diachronous author self-citation rate in urology is higher compared to general medical literature but similar to other surgical subspecialties. It may depend on the study design and the number of authors in the paper. Though generally viewed negatively in the scientific community, self-citations may be perfectly healthy. It is the intention behind self-citations that holds more importance but may be difficult to measure. For a more comprehensive evaluation, future studies should look at the context in which self-citations were made.

## References

[1] Lancet T. The Lancet. Variation in surgery and surgical research. Lancet. 2013 Sep;382(9898):1071. 24075031 10.1016/S0140-6736(13)62006-1

[2] Kelly CD, Jennions MD. The h index and career assessment by numbers. 2006 Apr;21(4):167–170.Trends Ecol. Evol. 16701079 10.1016/j.tree.2006.01.005

[3] Bartneck C, Kokkelmans S. Detecting h-index manipulation through self-citation analysis. Scientometrics. 2011;87(1):85–98. 21472020 10.1007/s11192-010-0306-5 PMC3043246

[4] Ioannidis JPA. A generalized view of self-citation: direct, co-author, collaborative, and coercive induced self-citation. J Psychosom Res. 2015 Jan;78(1):7–11. 25466321 10.1016/j.jpsychores.2014.11.008

[5] Lawani SM. On the heterogeneity and classification of author self-citations. 2007 Sep;33(5):281–284.J. Am. Soc. Inf. Sci.

[6] Kulkarni AV, Aziz B, Shams I, et al. Author self-citation in the general medicine literature. PLoS ONE. 2011 Jun;6(6):e20885. 21698195 10.1371/journal.pone.0020885 PMC3116850

[7] Swanson EW, Miller DT, Susarla SM, *et al*. What effect does self-citation have on bibliometric measures in academic plastic surgery? Ann. Plast. Surg. 2016;77(3):4. 26101989 10.1097/SAP.0000000000000585

[8] Tolisano AM, Song SA, Cable BB, *et al*. “Author self-citation in the otolaryngology literature,” *Head Neck Surg*., p. 5. 10.1177/0194599815616111 26556466

[9] Livas C, Delli K, Pandis N. Author self-citation in orthodontics is associated with author origin and gender. Prog Orthod. 2021 Jan;22(1):DOI:10.1186/s40510-020-00348-y PMC7788150 33409710

[10] Dessouky R, Zhang L, Wadhwa V, et al. Self-citations in musculoskeletal radiology: frequency and pattern analysis. Acta Radiol. 2019 Nov;60(11):1490–1495. 30845816 10.1177/0284185119836215

[11] Svider PF, Pashkova AA, Choudhry Z, *et al*. Comparison of scholarly impact among surgical specialties: an examination of 2429 academic surgeons. Laryngoscope. 2013;123(4):884–889. 23417821 10.1002/lary.23951

[12] Gami AS, Montori VM, Wilczynski NL, et al. Author self-citation in the diabetes literature. 3. 10.1503/cmaj.1031879 PMC421720 15210641

[13] Reiter O, Mimouni M, Mimouni D. Analysis of self-citation and impact factor in dermatology journals. Int J Dermatol. 2016 Sep;55(9):995–999. 26695728 10.1111/ijd.13193

[14] Sundaram K, Warren J, Anis HK, et al. Publication integrity in orthopaedic journals: the self-citation in orthopaedic research (SCOR) threshold. 2020 May;30(4):629–635.Eur. J. Orthop. Surg. Traumatol. 31858259 10.1007/s00590-019-02616-y

[15] Long X, Huang J-Z, Ho Y-S. A historical review of classic articles in surgery field. Am J Surg. 2014 Nov;208(5):841–849. 25167972 10.1016/j.amjsurg.2014.03.016

[16] Ahmad SS, Ahmed A, Exadaktylos A, *et al*. Systematic review on citation classics in minimally invasive gastrointestinal surgery. J. Minimal Access Surg. 2018;14(4):265. 10.4103/jmas.JMAS_149_18 PMC6130179 30106025

[17] Aksnes DW. A macro study of self-citation. Scientometrics. 2003 Feb;56(2):235–246.

[18] Mishra S, Fegley BD, Diesner J, et al. Self-citation is the hallmark of productive authors, of any gender. PLoS ONE. 2018 Sep;13(9):e0195773. 30256792 10.1371/journal.pone.0195773 PMC6157831

[19] Fassoulaki A, Papilas K, Paraskeva A, et al. Impact factor bias and proposed adjustments for its determination. Acta Anaesthesiol Scand. 2002;46(7):902–905. 12139549 10.1034/j.1399-6576.2002.460723.x

